# Preservation of the Blood Brain Barrier and Cortical Neuronal Tissue by Liraglutide, a Long Acting Glucagon-Like-1 Analogue, after Experimental Traumatic Brain Injury

**DOI:** 10.1371/journal.pone.0120074

**Published:** 2015-03-30

**Authors:** Jakob Hakon, Karsten Ruscher, Bertil Romner, Gregor Tomasevic

**Affiliations:** 1 Laboratory for Experimental Brain Research, Wallenberg Neuroscience Center, Lund University, BMC A13, Lund, Sweden; 2 Department of Neurosurgery, Copenhagen University Hospital, Risgshospitalet, Denmark; 3 Department of Neurosurgery, University Hospital of Lund, Lund, Sweden; University of Lancaster, UNITED KINGDOM

## Abstract

Cerebral edema is a common complication following moderate and severe traumatic brain injury (TBI), and a significant risk factor for development of neuronal death and deterioration of neurological outcome. To this date, medical approaches that effectively alleviate cerebral edema and neuronal death after TBI are not available. Glucagon-like peptide-1 (GLP-1) has anti-inflammatory properties on cerebral endothelium and exerts neuroprotective effects. Here, we investigated the effects of GLP-1 on secondary injury after moderate and severe TBI. Male Sprague Dawley rats were subjected either to TBI by Controlled Cortical Impact (CCI) or sham surgery. After surgery, vehicle or a GLP-1 analogue, Liraglutide, were administered subcutaneously twice daily for two days. Treatment with Liraglutide (200 μg/kg) significantly reduced cerebral edema in pericontusional regions and improved sensorimotor function 48 hours after CCI. The integrity of the blood-brain barrier was markedly preserved in Liraglutide treated animals, as determined by cerebral extravasation of Evans blue conjugated albumin. Furthermore, Liraglutide reduced cortical tissue loss, but did not affect tissue loss and delayed neuronal death in the thalamus on day 7 post injury. Together, our data suggest that the GLP-1 pathway might be a promising target in the therapy of cerebral edema and cortical neuronal injury after moderate and severe TBI.

## Introduction

Cerebral edema is a common life-threatening complication after traumatic brain injury (TBI) and represents a serious clinical problem due to the lack of specific treatments [1]. Cerebral edema promotes elevation of the intracranial pressure, and subsequently limits cerebral blood flow and tissue oxygenation leading to neuronal death and poor clinical prognosis [2]. The pathophysiological mechanisms behind the development of cerebral edema are multifaceted. It is generally agreed that both vasogenic and cytotoxic components contribute to edema in patients with moderate to severe TBI [3]. Vasogenic edema is caused by an inflammatory cascade with increased permeability of the blood-brain barrier (BBB) and movement of molecules and water to the brain interstitium [4].

Glucagon-like peptide-1 (GLP-1) is a gut-derived incretin hormone known for its effects on blood glucose homeostasis. GLP-1 interacts with a specific G-protein-coupled GLP-1 receptor (GLP-1R) that, besides being expressed on pancreatic cells, is also found on cerebral endothelial cells and various cells throughout the brain [5]. The half-life of endogenous GLP-1 is 1–2 minutes. Therefore, a long-acting GLP-1 analogue, Liraglutide, is used in the treatment of type 2 diabetes. Liraglutide binds selectively to the GLP-1R, and avoids being proteolyticly degraded by dipeptidyl peptidase-4 through binding to serum albumin [6]. Liraglutide has been reported to cross the BBB, and to have potent anti-inflammatory effects on cerebral endothelial cells and astrocytes [7–9]. Additionally, GLP-1R stimulation has been suggested to mediate neuroprotective effects in experimental stroke [10–13], Parkinson’s disease, and Alzheimer’s disease [14,15] and to promote neurogenesis [16].

The purpose of this study was to investigate whether treatment with Liraglutide improves neurological outcome in a rat model of moderate and severe TBI. We hypothesized that Liraglutide may attenuate BBB disruption and reduce neuronal loss after TBI.

## Materials and Methods

### Ethics Statement

All animal procedures were approved by the Malmö-Lund ethical committee (ethical permit number: M 19–12) and reported according to the ARRIVE guidelines. Adult male Sprague-Dawley rats (300–400 g, Charles River) were used. The rats were housed in standard laboratory cages (2 rats per cage) and in a reverse light-cycle. Behavioral tests were performed in the dark period when the rats were active. The rats had free access to food and water, and were kept in this environment for a minimum of 5 days before the experiments were performed.

### Experimental design

**Fig 1:**
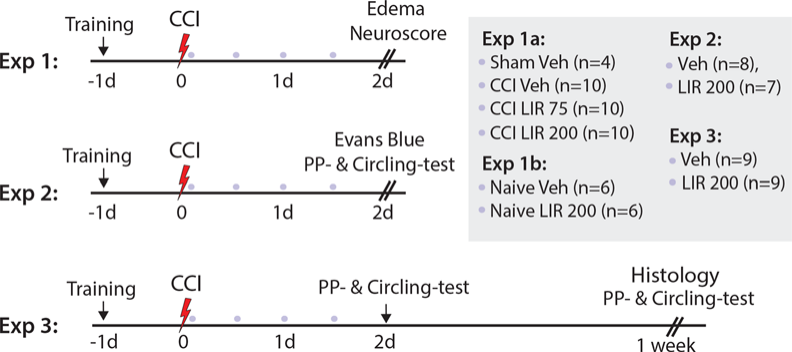
Experimental outline. Flow diagram of the three experiments. The colored dots indicate treatment with a subcutaneous dose of Liraglutide 75 μg/kg (LIR75), Liraglutide 200 μg/kg (LIR200) or vehicle. Abbreviations: Controlled cortical impact (CCI), days (d), experiment (exp), Paw-placement test (PP-test).

Three separate experiments were conducted. A total of 79 rats were included in the experiments (for experimental outline see Fig. 1).

For all experiments the first dose was given 10 min after Controlled Cortical Impact (CCI), and thereafter at 12, 24 and 36 hours after CCI. Investigators were blinded to injury status and pharmacological treatment of the rats.

*Experiment 1.a*: Thirty rats were subjected to CCI and randomized to subcutaneous treatment with either Liraglutide at a dose of 75 μg/kg (n = 10) or 200 μg/kg (n = 10), or vehicle (phosphate buffered saline, n = 10). Four rats were sham-operated. Neuroscore and brain edema were assessed 48 hours after CCI. Experiment 1.b: To assess the effect of Liraglutide on normal brain water content naive rats were injected with vehicle (n = 6) and Liraglutide 200 μg/kg (n = 6), and edema and Neuroscore was assessed 48 hours after CCI.

*Experiment 2*: The effect of vehicle (n = 8) and Liraglutide treatment (200 μg/kg; n = 7) twice daily on BBB integrity was tested by Evans blue extravasation 48 hours after CCI, together with circling test and paw-placement test.

*Experiment 3*: The effect of vehicle (n = 9) and Liraglutide 200 μg/kg (n = 9) on cortical lesion size and delayed thalamic neuronal death was determined 7 days after CCI. Results from the circling test and paw-placement test were recorded on day 2 and 7 post CCI.

### Controlled cortical impact (CCI)

Experimental TBI is induced by CCI, a clinically relevant model of moderate and severe TBI [17]. The rats were anesthetized with sodium pentobarbital (60 mg/kg, intraperitoneal injection) and positioned in a stereotactic frame. This specific anesthetic regimen has minimal effects on cerebral metabolism and cerebral blood glucose concentration [18]. Following a midline scalp incision, a 5 mm craniotomy was performed centrally over the left parietal bone. The bone flap was removed and the dura was kept intact. A cortical impact of approximately 200 milliseconds (msec) was induced with a 5 mm pneumatically driven piston at a velocity of 4 m/s and a penetration depth of 3 mm as previously described [19,20]. Subsequently, the scalp incision was closed, and the rats were allowed to recover. Rectal temperature was recorded and maintained at 37±1°C by a heating pad throughout the surgical procedure and for a minimum of 3 hours post-injury until the rats were fully active. Sham-operated rats were exposed to the same surgical and peri-surgical procedures except the cortical impact.

### Evaluation of neurological function

#### Composite Neuroscore

Sensorimotor function was evaluated by a composite Neuroscore adapted from Hunter et al. [21]. The composite Neuroscore consists of eight individual tests, each rated on an integral scale: general motility when placed on a platform (maximum 3 points), circling test (maximum 3), paw placement back to bench when slid over an edge (maximum 4), forepaw reaching (maximum 2), the rats ability to lift itself up when hanged by their forepaws on a horizontal bar (maximum 3), the ability to climb a 45° inclined plane without foot slips (maximum 3), contralateral reflex when lifted by the tail (maximum 1) and grip strength (maximum 2). A composite Neuroscore was calculated, giving a maximum score of 21 points. Rats were tested the day before injury to ensure that they were all able to obtain the maximum score in each subtest.

#### Circling test

This test assesses the function of front and hind limbs. It is recorded whether the rat turns towards the paretic side spontaneously or not, when lifted by its tail, and when pulled along the floor by its tail. For each sub-test, the rats got one point when showing symmetrical behavior [21,22].

#### Paw-placement test

The paw placement test was performed in order to assess tactile and proprioceptive sensory function [23,24]. The rats were placed on a tabletop and moved laterally towards the edge, until the limbs closest to the edge lost contact with the table surface. The deficit for each fore- and hindpaw was then evaluated as follows: When a paw was quickly moved back to the surface, the rat was assigned 1 point. If a paw was not moved back to the surface but inwards towards the side of the table edge and supinated, it scored 0.5 points. A persistent free-hanging limb was assigned 0 points. The score for each limb was combined to give a maximum score of 4 points. During the tests, care was taken that the head was straight at all times, and that the rats were not able to see their free-hanging limbs.

### Brain Water Content

Brain water content was evaluated using the wet/dry-weight method, a reliable measure of posttraumatic brain edema [25]. Forty-eight hours after CCI, rats were anesthetized with 4% isoflurane (Baxter, Miami, FL) and decapitated. The brains were quickly removed from the cranium and cut into 6 mm coronal sections including the lesioned area. The tissue slab was quickly divided into ipsi- and contra-lateral cortex, hippocampus and thalamus. The samples for each region were placed on aluminum foil and weighed to obtain wet-weight. Samples were then dried at 90°C for 72 hours and reweighed for dry-weight. The percentage of water content was calculated using the following equation: ([wet weight—dry weight]/wet weight) x 100.

### Blood-Brain Barrier integrity

The degree of BBB disruption was determined using the Evans blue dye extravasation as described previously [26]. Rats were anesthetized with pentobarbital as described above. Two percent Evans blue dissolved in phosphate-buffered saline (PBS) was injected intravenously (5 ml/kg). After one hour, the rats were perfused transcardially with a minimum of 400 ml saline through the left ventricle until colorless perfusion fluid was obtained from the right atrium. The brain was removed and sectioned into left and right hemispheres. The cerebellum was collected as an internal control. Each sample was then placed in 2 mL formamide and incubated at room temperature for 48 hours. The amount of Evans blue in the supernatant was measured by absorbance of the supernatant using a double beam spectrophotometer (U2800 Hitachi, Japan). A linear standard curve was made based on Evans blue external standards (0.47 to 37.5 ng/ml). The tissue was then dried at 95°C for 5 days. Based on the standard curve the amount of Evans blue was quantified as μg of Evans blue per mg dry weight of brain tissue.

### Histochemical processing

Rats were anesthetized with isoflurane and transcardially perfused with PBS followed by 4% paraformaldehyde (PFA) in PBS. The brains were incubated over night in 4% PFA and thereafter transferred to a 25% sucrose solution for a minimum of 48 hours. The brains were cut into 40 μm coronal sections using a sliding microtome and one section was collected every 1 mm for measurement of lesion size.

### Evaluation of neuronal tissue loss

The lesion size was assessed at day 7, at which point it is considered to be matured [27]. Free-floating sections were washed three times in PBS and quenched (3% H2O2 and 10% methanol) for 15 minutes. After blocking in 5% normal donkey serum and 0.25% Triton X-100 in PBS for 60 minutes, the sections were incubated in blocking solution containing a monoclonal mouse anti-NeuN antibody (1:1500, Millipore, Hampshire, UK) at 4°C overnight. After primary antibody incubation, the sections were rinsed and incubated with a biotinylated secondary donkey anti-mouse antibody (1:400, Vector Laboratories, USA) for 60 minutes in blocking solution. Visualization was done using a Vectorstain ABC kit (PK-6100, Vectorlab) and 3,3-diaminobenzidine/H_2_O_2_ (DabSafe, Saveen Werner, Sweden). Stained sections were mounted on glass. The slices were scanned, and the cortical, thalamic and hemispherical lesion volumes were determined using ImageJ by subtracting the area of healthy tissue on the contralateral side from that of the ipsilateral side for each section [28].

### Evaluation of delayed neuronal death

Floating sections were stained with Fluoro-Jade C (FJC) in order to evaluate degenerating neurons [29]. Sections corresponding to 2.8 mm posterior of bregma were mounted and incubated with 1% NaOH in 80% alcohol for 5 min, 2 min in 70% alcohol, and 2 min in distilled water. The sections were then incubated in a 0.06% potassium permanganate solution for 10 min with agitation, later washed, and subsequently incubated in a solution of 0.0005% FJC (Millipore) in 0.1% acetic acid for 20 min. Finally, the slides were dried and coverslipped. Images were obtained on a LSM510 confocal microscope (Zeiss, Germany). A blinded researcher captured 5 images within the thalamic area guided by the DAPI-stain. For analysis ImageJ software with Point Picker plugin was used. Data are presented as an average of the 5 areas of interest.

### Blood glucose

Blood glucose levels were measured with a Contour glucometer (Bayer, Germany). An average value of two consecutive measurements was recorded.

### Statistical analysis

Analyses were performed using Prism Graph pad 6. All data are presented as mean ± SEM if nothing else is stated. P<0.05 was defined as statistically significant. Statistical differences between two groups were determined using students T-test. Neuroscore data were analyzed using a non-parametric Kruskal-Wallis test followed by Dunn’s test for multiple comparisons. Effects on subregional water content were tested using a two-way analysis of variance (ANOVA) test with treatment group and brain region as main variables, followed by Tukey’s *post hoc* test for multiple comparisons. Effects of treatment dose on total water content (experiment 1), and differences in temperature, blood glucose and body weight between treatment-groups, were analyzed with one-way ANOVA followed by Dunnet’s post-hoc test for each time point. Group sizes were decided before conducting any experiments based on previous investigations [8,26,30].

## Results

### Effect on physiological parameters

Overall, there were no effects of Liraglutide treatment on body temperature (Table 1). Mean blood glucose in sham animals ranged from 5.6–6.0 mmol/l throughout the experiment. At the 30 minute timepoint both treatment groups were slightly hyperglycemic, however, at this time point Liraglutide treated animals had significantly lower blood glucose compared to vehicle (Liraglutide: 6.6 ± 0.16 mmol/l, vehicle: 7.1 ± 0.17 mmol/l; p<0.05). At 12 hours, Liraglutide-treated animals had significant higher blood glucose than vehicle-treated animals (Liraglutide: 6.35 ± 0.28 mmol/l; vehicle: 5.3 ± 0.14 mmol/l; p<0.05), but did not differ significantly from sham-treated animals. Body weight % of baseline was significantly lower at all time points after CCI in Liraglutide-treated compared to vehicle-treated animals. However, at day 7, Liraglutide-treated animals had recovered their body weight to what it was before the injury (Table 1).

**Table 1:** Summary of blood glucose, core temperature and weight %.

	Core Temperature			
	*12 hours*	*24 hours*	*26 hours*	*48 hours*
Sham	38.4 ± 0.19	37.5 ± 0.34	38.0 ± 0.00	38.1 ± 0.19
Vehicle	37.7 ± 0.21	38.3 ± 0.15	38.2 ± 0.13	38.4 ± 0.15
LIR 75	37.7 ± 0.10	38.0 ± 0.17	38.1 ± 0.14	38.1 ± 0.07
LIR 200	37.3 ± 0.19	37.6 ± 0.15*	37.9 ± 0.08	38.1 ± 0.15
	**Blood glucose**			
	***30 min***	***12 hours***	***24 hours***	***48 hours***
Sham	-	6.00 ± 0.08	5.38 ± 0.14	5.98 ± 0.30
Vehicle	7.1 ± 0.17	5.48 ± 0.20	5.16 ± 0.18	5.96 ± 0.29
LIR 75	-	5.32 ± 0.14	4.82 ± 0.16	5.57 ± 0.18
LIR 200	6.6 ± 0.16*	6.35 ± 0.28*	5.47 ± 0.18	5.33 ± 0.18
	**Weight % of baseline**			
	***12 hours***	***24 hours***	***48 hours***	***7 days***
Sham	98.4 ± 0.82	97.1 ± 1.10	95.8 ± 1.32	-
Vehicle	96.3 ± 0.40	91.6 ± 0.36	94.4 ± 0.44	103 ± 0.87
LIR 75	94.7 ± 0.34*	94.3 ± 0.39*	89.3 ± 0.55*	-
LIR 200	93.1 ± 0.43*	90.5 ± 0.43*	87.7 ± 0.43*	96.1 ± 0.71*
Naive vehicle	100 ± 0.49	100 ± 0.50	101 ± 0.80	-
Naive LIR 200	93.7 ± 0.49*	90.5 ± 0.61*	91.1 ± 1.08*

* Indicates significant difference from vehicle.

### Liraglutide treatment reduces CCI induced neurological deficits

**Fig 2:**
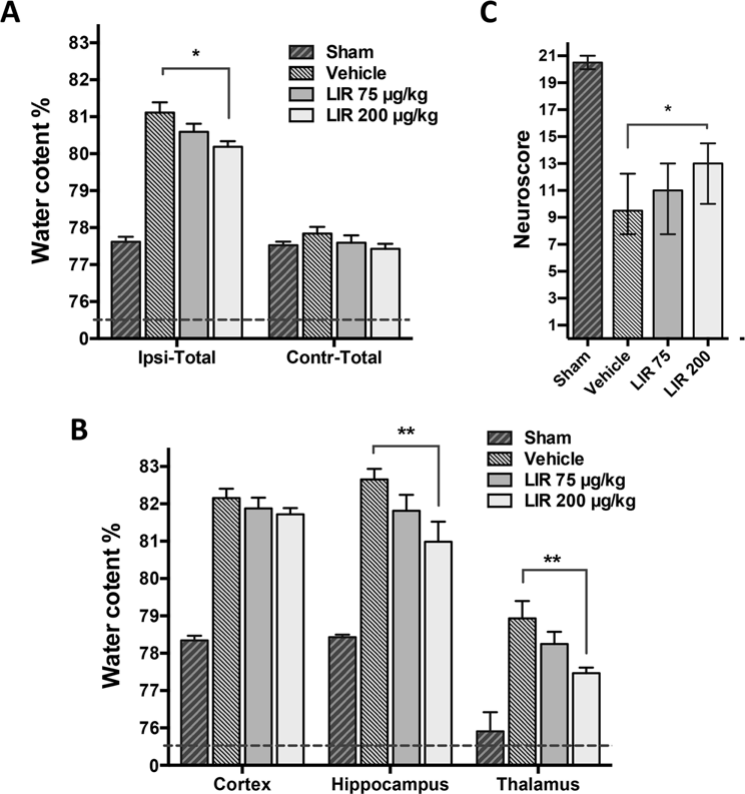
Effect of Liraglutide on brain water content and Neuroscore after TBI. A: Ipsilateral total water content of the 6 mm coronal sample area (Ipsi-Total) and corresponding contralateral region (Ctrl-Total) 48 hours following controlled cortical impact (CCI). Total water content increased significantly in vehicle treated animals compared to Sham. However, water content was mitigated significantly in animals treated with Liraglutide 200 μg/kg BID. B: Subregion water content of contralateral and ipsilateral areas 48 hours after CCI. Liraglutide 200 μg/kg BID significantly mitigated cerebral water content in the ipsilateral hippocampus and thalamus after CCI. C: Composite Neuroscore test was calculated through a battery of six sub-tests 48 hours after CCI, with a score of maximum 21 points. Values in A and B are means ± SEM. *:p<0.05, **:p<0.01. Values in C are presented as median and error bars indicate the 25^th^ and 75^th^ percentile. Abbreviations: LIR75—Liraglutide 75 μg/kg; n = 10, LIR200—Liraglutide 200 μg/kg; n = 10, vehicle n = 10 and Sham n = 4.

At 48 hours post-CCI, injured vehicle treated animals demonstrated significant sensorimotor impairments when compared with sham-operated rats (p<0.001). Liraglutide treatment (200 μg/kg) significantly improved composite Neuroscore compared to vehicle treated rats (p<0.05, Fig. 2B).

**Fig 3:**
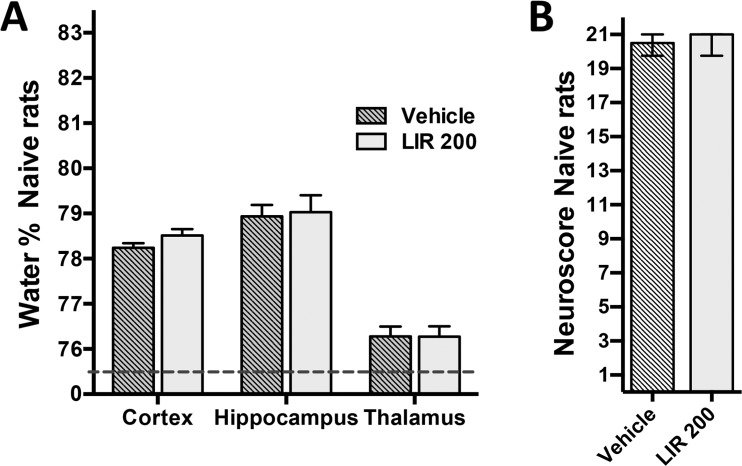
Effect of Liraglutide on normal brain water content and Neuroscore. Water content in the uninjured naive brain was unaffected by Liraglutide 200 μg/kg 48 hours after CCI. Values are means ± SEM. B: There was no effect of Liraglutide on Neuroscore in naive animals. Values are median with 25^th^ and 75^th^ percentile.

Liraglutide 75 μg/kg did not significantly improve Neuroscore after CCI. Individual scores for paw placement and circling tests in experiment 1 were significantly higher in the group treated with Liraglutide 200 μg/kg (data not shown). There was no effect of Liraglutide 200 μg/kg on Neuroscore when given to naive rats (Fig. 3B).

**Fig 4:**
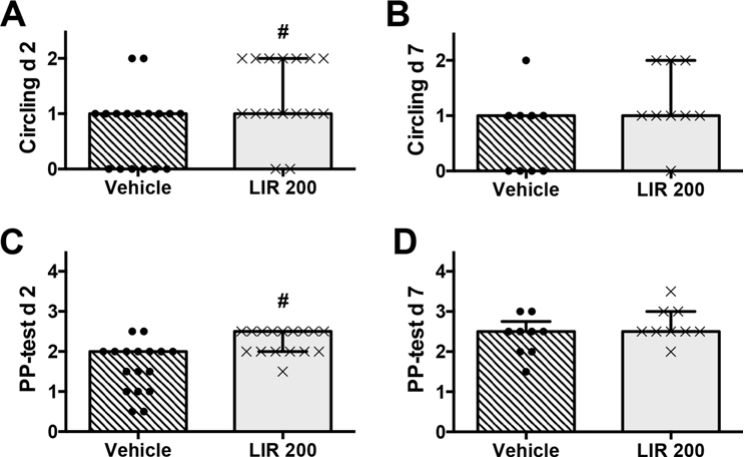
Paw placement and Circling test (experiment 2 and 3). Performance of vehicle- and Liraglutide-treated rats in Circling test 2 days (A) and 7 days (B) after controlled cortical impact (CCI), and limb-placing ability for 2 days (C) and 7 days (D) after CCI. The normal score before injury is 2 for circling test and 4 for paw placement test. Values are presented as median and error bars indicate the 25^th^ and 75^th^ percentile, #:p<0.05.

In experiment 2 and 3, the circling test showed severe sensorimotor impairment in vehicle-treated rats 2 and 7 days after CCI (Fig. 4A and 4B). This impairment was significantly attenuated by Liraglutide (200 μg/kg) at day 2 (p<0.01) but not at day 7 after CCI. Likewise, Liraglutide-treated animals showed a significantly better improvement than vehicle-treated animals only at day 2 after CCI (p<0.05, Fig. 4C). Due to spontaneous recovery in paw placement both in vehicle and Liraglutide group no differences between the treatment groups were observed on day 7 after CCI (Fig. 4D). Together, these results demonstrate that administration of Liraglutide improves sensorimotor outcome 48 hours after CCI.

### Liraglutide treatment ameliorates cerebral edema

As shown in Fig. 2A, total water content of the brain tissue sample including all three regions studied was 77.6 ± 0.3% in sham-operated animals. In vehicle-treated rats subjected to CCI, water content increased to 81.1 ± 0.3%. Treatment with Liraglutide (200 μg/kg) led to a total water content of 80.19 ± 0.2%. This represents a significant mitigation of total water content elevation, i.e. edema, by 26% compared to vehicle treated rats (p<0.05). Similarly, subregion analysis of the hippocampus and thalamus representing the pericontusional areas revealed that Liraglutide significantly mitigated hippocampal edema by 39% (p<0.01, vehicle: 82.6 ± 0.3% vs. Liraglutide: 81.0 ± 0.6%; Sham: 78.4 ± 0.2%) and thalamic edema by 48% (p<0.01, vehicle: 78.9 ± 0.5% vs. Liraglutide: 77.5 ± 0.1%; Sham: 75.9 ± 0.1%) (Fig. 2C). There was no significant effect of Liraglutide on water content in the ipsilateral cerebral cortex region. The reduction of brain water content in animals treated with the lower Liraglutide dose (75 μg/kg) was insignificant. Liraglutide did not influence water content in normal brain tissue when given to naive animals (Fig. 3A).

### Liraglutide treatment reduces BBB permeability

**Fig 5:**
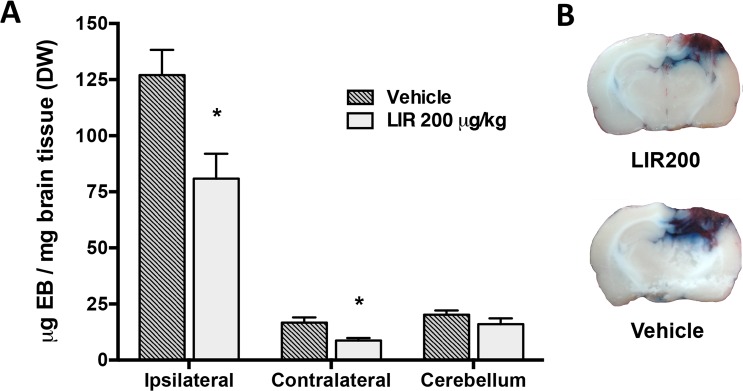
Evans blue exudation. A: Analysis of Evans Blue extravasation (μg/mg of dry brain tissue) 48 hours after CCI in rats. Treatment with Liraglutide 200 μg/kg BID significantly reduced Evans Blue extravasation in both hemispheres. B: Illustration of coronal sections through the contusion center illustrates Evans blue distribution throughout the rat brain. Evans blue extravasation is particularly prominent in the pericontusional cortex, hippocampus and upper thalamus. Values are mean ± SEM. *:p<0.05.

To further examine the effect of Liraglutide (200 μg/kg) on edema, we used Evans Blue as a marker of Albumin extravasation (Fig. 5B). The Evans blue content of the ipsilateral hemisphere of vehicle-treated animals (127 ± 11 μg EB/mg) was significant higher than the cerebellar control 48 hours after CCI (20.2 ± 1.9 μg EB/mg; p<0.0001). Liraglutide led to a significant reduction in Evans blue extravasation in the ipsilateral hemisphere (80.9 ±11 μg EB/mg; p<0.05) (Fig. 5A). The contralateral hemisphere had significantly less Evans blue in Liraglutide-treated animals (8.7 ± 1.2 μg EB/mg) compared to vehicle-treated animals (16.7 ± 2.7 μg EB/mg; p<0.05).

### Liraglutide reduced cortical lesion size but had no effect on delayed thalamic neuronal death

**Fig 6:**
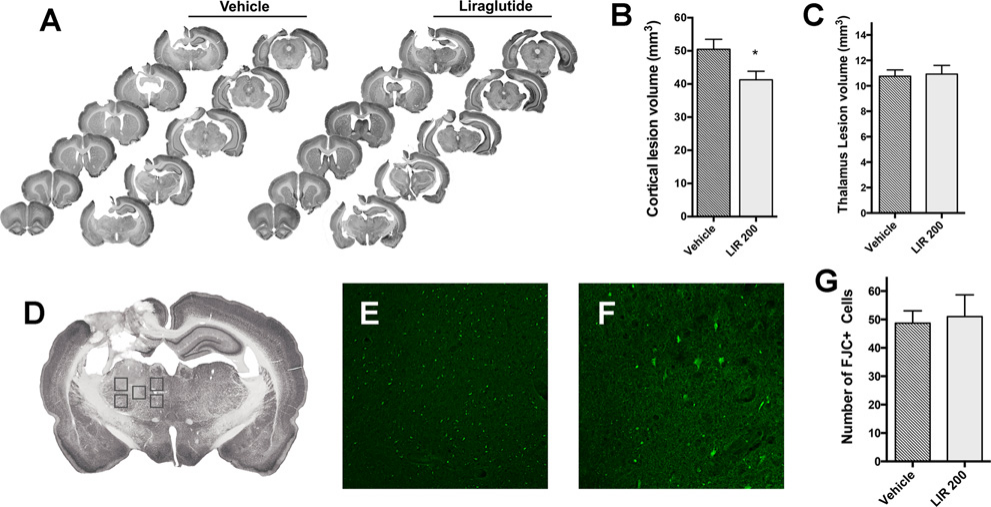
Effects on lesion volume and delayed neuronal death 7 days post-injury. A: Illustration of representative lesions by NeuN stained coronal sections from rats treated either with vehicle or Liraglutide for two days. The successive coronal sections range from +2.2 to -6.8 mm from bregma. B: Calculated cortical lesion volume (mm^3^). C: Calculated lesion volume in the ipsilateral thalamus (mm^3^). D: NeuN stained coronal section -2.8 mm from bregma illustrating the 5 regions of interest (ROI) chosen for investigation of FJC+ cells within the thalamus. Each ROI represents a counting frame at 10X. E: Demonstration of counting frame with FJC+ cells 7 days after CCI. F: Degenerating neurons shown at 20X. G: The average number of FJC+ cells for the 5 ROI’s was unaffected by Liraglutide treatment for the first 2 days post injury. *:p<0.05. Values are means ± SEM.

At day 7, a large lesion was apparent involving the cerebral cortex and subcortical structures including the corpus callosum, hippocampus and thalamus. The lesion was generally visible at +1.2 mm to -6.8 mm from bregma (Fig. 6A). Fig. 6B shows a significant reduction in cortical lesion size in Liraglutide-treated rats (41.3 ± 2.6 mm^3^) compared to vehicle treated animals (50.5 ± 3 mm^3^; p<0.05) at 7 days. There was no effect of Liraglutide on lesion size in the thalamus (Fig. 6C). Additionally, when estimating lesion size for the whole ipsilateral hemisphere, Liraglutide-induced reduction in lesion size was not significant compared to vehicle (data not shown). To assess degenerating neurons one week after CCI we performed FJC stainings of the sections collected at day 7 post injury. FJC positive (FJC+) cells were present in the ipsilateral thalamus. We found no difference in number of FJC+ cells between vehicle and Liraglutide-treated animals in any of the individual five thalamus ROI’s (Fig. 6C-E).

## Discussion

In this study we investigated the effect of post-TBI Liraglutide treatment on sensorimotor deficits, cerebral edema, BBB integrity, lesion volume, and delayed neuronal death.

### Neurological function

TBI is associated with impairment of a wide range of neurobehavioral modalities including cognitive, emotional and sensorimotor function [31]. An emerging body of evidence indicates that treatment with Exendin-4 (Ex-4), a systemic GLP-1 agonist, abolishes cognitive deficits in rodent models of mild and moderate TBI [32–35]. In more detail, treatment with Ex-4 starting both 2 days before and 1 hour after mild concussive TBI improved memory function at day 7 and day 30 [33]. Additionally, in mild TBI, Tweedie et al. found that improvement in recognition memory by Ex-4 pretreatment was related to reduced changes in hippocampal genes related to Alzheimer’s disease [32]. Similar studies have been conducted in rats where Ex-4 administration 30 minutes after moderate fluid-percussion TBI improved memory function when assessed using a water maze test. In the above mentioned experiment, Ex-4 treatment was ended at a minimum of 2 days before any behavioral test to avoid confounding effects of Ex-4 on cognitive tests [32]. In the present study, the last dose was given 36 hours after TBI, that is, 12 hours before conducting any neurological test. However, as shown in Fig. 3B, this regime had no adverse effect on sensorimotor outcome in normal rats.

It has been reported that Liraglutide effectively improves sensorimotor function 24–72 hours post injury when, assessed by a variety of neuroscore tests, in different models of experimental stroke [8,13]. However, to our knowledge, the effect of GLP-1 receptor agonists on sensorimotor outcome after TBI has not previously been studied. Here we showed that Liraglutide treatment improved sensorimotor function 48 hours after TBI. However, this effect was attenuated on day 7, likely as a result of spontaneous functional recovery related to plasticity processes in the lesioned brain [36]. Cerebral edema peaks within the first two days after CCI, and is considered a primary causal factor of neuronal injury and sensorimotor deficits after moderate and severe TBI [25,37]. Therefore, it is likely that the observed positive effects of Liraglutide on neurological function 48 hours after CCI are to some extent related to anti-edema effects.

### Edema and BBB

The levels of cerebral water content, and the regional differences in water content between cortical, hippocampal and thalamic areas that we report in this study, are in line with previous reports [26,38]. Liraglutide (200 μg/kg) BID significantly mitigated TBI induced water content increase in the hippocampus and thalamus by 39% and 48%, respectively. In contrast, Liraglutide did not significantly reduce edema in the cortical region. This might be caused by a local low Liraglutide delivery or different edema pathology in the contusion core, which has been shown to be markedly hypoperfused and sparsely vascularized [39]. In addition to cerebral edema, we found that Liraglutide markedly reduced Evans Blue extravasation in the ipsilateral and contralateral hemisphere. This indicates that GLP-R stimulation prevents endothelial barrier dysfunction, and suggests that the reduction in tissue water content, at least partially, is due to reduced vasogenic edema [26]. From Fig. 5B it can be discerned that Evans blue mainly is located pericontusionally in the contralateral hemisphere and to some extent in the contralateral cingulate cortex adjacent to the contusion. The increased Evans Blue extravasation in the uninjured contralateral hemisphere is likely due to the high magnitude of the injury.

Recruitment of neutrophils to the brain parenchyma plays a key role in edema formation [30,40–42]. Together with resident microglial cells, neutrophils mediate BBB disruption through release of glutamate, reactive oxygen species (ROS), and proteases such as matrix metallo-proteinases [43–45]. Glutamate and ROS also act in a toxic manner on parenchymal cells, thereby contributing to neuronal death and cytotoxic edema [4]. Several studies have pointed out an anti-inflammatory role of GLP-1 in acute brain injury, for instance Exendin-4 reduced IBA-1 positive microglia activation in experimental model of global ischemia and in stroked diabetic rats subjected to experimental stroke [10,11]. In addition, it has been shown that Liraglutide reduces cerebral edema formation in a mouse model of hemorrhagic stroke [8]. This effect was related to an anti-inflammatory effect on cerebral endothelial cells through reduced expression of ICAM-1 and subsequently a reduced recruitment of neutrophils to the brain parenchyma. Likewise in diabetic vascular disease, GLP-1 has anti-inflammatory effects on cardiovascular endothelial cells [46]. Liraglutide also reduced the expression of ROS and various adhesion molecules in cultures of human endothelial cells [47,48]. Thus, it is likely that the reduction in blood-brain barrier permeability and brain edema post-TBI demonstrated in the present study is related to the anti-inflammatory properties of Liraglutide.

### Protection of neuronal tissue

It is evident that improvement in motor and cognitive functions in models of experimental TBI is associated with a decrease in macroscopic cortical tissue loss [49,50]. In concordance with this we found that systemic treatment with Liraglutide for two days after CCI reduced the cortical lesion volume of cortical neuronal injury by approximately 20%. This finding supports previous studies showing that Liraglutide reduces cryogenic brain injury at 2 days [51], and implantation of GLP-1 producing stem cells reduced hippocampal neuron loss after CCI [52]. Both Liraglutide and Ex-4 have demonstrated neuroprotective properties after experimental stroke [10,12,53]. These in vivo findings are supported by in vitro studies reporting that GLP-1 reduces oxidative stress in cultures of human neuroblastoma cells [54,55], and glutamate-induced excitotoxicity in neuronal cultures derived from rats [33,55]. Finally, preservation of cortical tissue by Liraglutide after CCI may indirectly be related to edema alleviation, and subsequently increased perfusion and oxygen tension in pericontusional areas [2,56].

The time course of neuronal death differs between different regions after cortical trauma. Focal cortical injury is associated with delayed death of thalamic neurons likely due to retrograde damage of cortical projections from the thalamus [57]. We analyzed Fluoro-Jade C positive (FJC+) neurons in the thalamus 7 days after CCI. There was no effect of Liraglutide when administered for 2 days after injury on FJC+ neurons. This can be due to the magnitude of the injury or the fact that the treatment was not continued beyond two days after the injury.

### Glycemic control

Hyperglycemia occurs frequently after moderate and severe TBI [58,59], and is driven by a hypermetabolic sympathoadrenal stress response [60]. Several studies indicate that hyperglycemia in the early hours after severe TBI is associated with worse neurological outcome [58,61]. Hyperglycemia is believed to induce neurotoxicity through an increase of oxidative stress [62] and enhanced extracellular glutamate excitotoxicity [63]. In our study, both Liraglutide and vehicle treated animals were slightly hyperglycemic 30 minutes after the injury. However, Liraglutide treatment significantly reduced glucose levels at 30 minutes post injury.

Insulin is used worldwide for treatment of hyperglycemia in patients with severe TBI. However, several trials have reported that strict glucose control with insulin in patients with TBI is associated with variable degrees of hypoglycemia [64,65]. This is problematic since the injured brain is sensitive even to mild hypoglycemia [64]. On the other hand, it has been shown that GLP-1 enhances cerebral glucose transport and metabolism during hyperglycemia in humans [66], and that these effects are not present during hypoglycemia [67]. Furthermore GLP-1 reduces blood glucose mainly by stimulation of glucose dependent insulin synthesis, and suppression of glucagon secretion [5]. These effects of GLP-1 ceases when blood glucose reaches normal and hypoglycemic levels [68]. In accordance with this, treatment with Liraglutide did not cause hypoglycemia in our study. Therefore, our results support the hypothesis stated in a recent review by Greig et al., that GLP-1 might be considered as a safe therapy to diminish hyperglycemia after TBI [69].

### Dose considerations

In humans Liraglutide has a half-life of around 13 hours, and is administered once daily in doses up to 1.8 mg. To investigate the effect of Liraglutide on secondary injury after TBI, we chose a dosing regime with two daily injections, starting immediately after injury, to compensate for a known shorter half-life of Liraglutide in rats (4 hours) compared to humans [70]. The lowest dose used in this study was 75 μg/kg BID, which is comparable with the currently used dose for antidiabetic treatment in humans [71]. The highest treatment dose used in this study (200 μg/kg BID), is somewhat higher than what is currently used for antidiabetic treatment. This dose, however, has been used previously in several rodent experiments [72,73]. In our experiments, both doses of Liraglutide resulted in a temporary reduction in body weight. This is a known side effect of Liraglutide [74], mediated by reduced appetite and gastric emptying [6]. Moreover, in a clinical trial by Astrup et al.[75] and recently in a phase III clinical trial [76], a high dose of 3 mg was effective at inducing weight loss and was well tolerated. Thus, even though 200 μg/kg BID currently has less translational value, it is likely that higher doses will be in clinical use for other conditions within the near future.

## Conclusions

Treatment with Liraglutide reduced cerebral edema and cortical neuronal tissue loss, and improved BBB integrity and sensorimotor outcome after experimental TBI. These novel findings extend prior research with GLP-1 agonists in TBI and experimental stroke. Further experiments are needed to delineate the exact mechanisms by which Liraglutide decreases cerebral edema and cortical tissue loss after TBI. Furthermore, it will be important to elucidate if longer treatment periods will provide better neuroprotective effect and functional outcome after CCI, and whether a later treatment onset is efficacious.

Prevention of cerebral edema and neuronal injury remains an important challenge in TBI patients. This study indicates that GLP-1R stimulation is a promising target in terms of sensorimotor sequelae after TBI. Given that several GLP-1 agonists, including Liraglutide, are already approved for clinical use and are well tolerated, further exploration of these effects is warranted.
